# A Novel Edible Coating Produced from a Wheat Gluten, *Pistacia vera* L. Resin, and Essential Oil Blend: Antimicrobial Effects and Sensory Properties on Chicken Breast Fillets

**DOI:** 10.3390/foods12122276

**Published:** 2023-06-06

**Authors:** Aykut Önder Barazi, Arzu Çağrı Mehmetoğlu, Osman Erkmen

**Affiliations:** 1 Food Engineering Department, Engineering Faculty, Gaziantep University, Gaziantep 27310, Turkey; barazi@gantep.edu.tr; 2 Food Engineering Department, Faculty of Engineering, Sakarya University, Sakarya 54187, Turkey; acagri@sakarya.edu.tr; 3 Department of Nutrition and Dietetics, Faculty of Health Sciences, Istanbul Arel University, Istanbul 34440, Turkey

**Keywords:** edible coating, *Salmonella*, *Listeria*, *Pistacia vera*, essential oil, chicken breast, resin

## Abstract

Antimicrobial edible coatings can eliminate the risk of pathogen contamination on the surface of poultry products during storage. In this study, an edible coating (EC) based on wheat gluten, *Pistacia vera* L. tree resin (PVR), and the essential oil (EO) of PVR was applied on chicken breast fillets (CBF) by a dipping method to prevent the growth of *Salmonella* Typhimurium and *Listeria monocytogenes*. The samples were packed in foam trays wrapped with low-density polyethylene stretch film and stored at 8 °C for 12 days to observe the antimicrobial effects and sensory properties. The total bacteria count (TBC), *L. monocytogenes*, and *S.* Typhimurium were recorded during storage. The samples coated with EC, containing 0.5, 1, 1.5, and 2% *v/v* EO (ECEO), showed significant decreases in microbial growth compared to the control samples. The growth of TBC, *L. monocytogenes*, and *S.* Typhimurium was suppressed by 4.6, 3.2, and 1.6 logs, respectively, at the end of 12 days on the samples coated with ECEO (2%) compared to the uncoated controls (*p* < 0.05). Coating with ECEO (2%) also preserved the appearance, smell, and general acceptance parameters better than uncoated raw chicken (*p* < 0.05) on the fifth day of storage. In grilled chicken samples, ECEO (2%) did not significantly change the appearance, smell, and texture (*p* > 0.05) but increased the taste and general acceptance scores. Therefore, ECEO (2%) can be a feasible and reliable alternative to preserve CBFs without adversely affecting their sensory properties.

## 1. Introduction

Food materials, especially perishable foods, must be protected by manufacturers and suppliers to meet consumer demands in terms of food safety and security. One of the main challenges in the food industry is keeping food safe and secure. In the commercial distribution of meat and poultry products, conventional methods, such as refrigeration, are insufficient to slow down deterioration during processing and storage. The three main mechanisms of meat and poultry deterioration are microbial spoilage, lipid oxidation, and enzymatic reactions (such as proteolytic and lipolytic enzymes seen in postslaughter and enzymes produced by microorganisms during storage) [1]. Chicken breast fillets (CBFs), which are the most popular poultry products to consume, are very susceptible to spoilage due to their native microflora. After slaughter, the native microflora of the poultry can contribute to the spoilage of poultry products if the meat is not properly stored or handled. Certain bacteria, such as *Pseudomonas*, *Brochothrix thermosphacta*, lactic acid bacteria, and *Enterobacteriaceae*, are particularly associated with spoilage [2]. Favorable intrinsic factors (i.e., high moisture, high pH, postslaughter chemical reactions, and enzymatic activity), poor handling, and adverse processing conditions (i.e., conditioned cuts, fluctuating temperatures, and cross contamination) may accelerate these deteriorations. Due to these factors, CBFs have a concise shelf life ranging between 5–8 days at refrigeration temperatures of 4–8 °C [1,3,4,5]. 

Aside from spoilage-caused deterioration, CBFs serve as a suitable medium for food pathogens when favorable conditions (high moisture content, optimal pH, available nutrients, handling processes) occur. Pathogenic and spoilage bacteria cause severe economic losses in the poultry industry and extensive health losses among customers each year. Among these microorganisms, 70% of foodborne illness outbreaks in the United States are attributed to *Salmonella* Typhimurium and *Listeria monocytogenes* [6]. With respect to Europe, according to the European Centre for Disease Prevention and Control (ECDC) report, the total numbers of reported cases of salmonellosis and listeriosis recorded in 2021 were 10,657 and 2243, respectively. While the group most affected by listeriosis was the elderly (65+ years old), those under 50 were most affected by salmonellosis [7]. 

To ensure the stability of food-safety parameters, traditional food-preservation techniques with suitable packaging have been used extensively in the food industry [8,9]. Classic packaging methods for food materials involve plastic (petroleum-based) materials due to their low price and flexible production characteristics [10]. However, plastic materials have several disadvantages due to their environmental and human health risks [11]. Traditional passive packaging techniques involving plastic materials keep food materials safe against extrinsic factors, such as physical damage, humidity, light, and oxygen. Nevertheless, they lack protection against water activity, enzymatic activity, chemical reactions, and microbial growth in food materials. The opposite mechanism developed to address this phenomenon is “active packaging”, which includes functional properties to control alterations and safety in food materials during storage. Active packaging can control the transfer of active components, such as the migration of antimicrobial substances inside packaging materials and food surfaces [12]. For instance, edible films and coatings with functional properties are often used for active packaging on the surfaces of food products [12,13].

### 1.1. Edible Films and Coatings

Edible films and coatings, defined as thin layers of material, provide a barrier to moisture, oxygen, and mass transfer on food products. Indeed, edible packaging can be used to encapsulate aroma compounds, antioxidants, antimicrobial agents, pigments, ions that stop browning reactions, or nutritional substances, such as vitamins [14]. Edible films and coatings have recently received considerable attention because of their advantages over synthetic packaging [15]. Over 90 patents and scientific papers concerning the manufacturing of edible packaging have been published since 1990.

Edible films and coatings typically contain three major compounds: proteins, polysaccharides, and lipids. Plasticizers can be added to film-forming solutions to enhance the physical properties of the film material [16]. Composite films contain various compounds (e.g., proteins, polysaccharides, and lipids) together with other functional ingredients (e.g., antimicrobials, antioxidants, enzymes, aroma compounds, etc.) to enhance the properties of films [12,14,15,17,18,19]. 

Edible coatings (ECs) may feature several functional properties besides their primary usage purposes. For example, they can carry antimicrobial agents that inhibit or kill pathogenic and spoilage microorganisms during storage. Many studies have examined antimicrobial agents used in ECs, such as benzoates, propionates, sorbates, parabens, acidifying agents (e.g., acetic, malic, citric, sorbic, lipophilic, and lactic acids), curing agents (e.g., sodium chloride and sodium nitrite), bacteriocins (nisin), lactoperoxidase systems, chitosan, and natural preservatives (e.g., essential oils, lysozyme, and liquid smoke) [20,21,22].

Incorporating essential oils (EOs) as natural and effective antimicrobial agents into ECs has gained significant interest in this research area. Studies have confirmed the antimicrobial effects of several plant-based EOs, such as oregano, thyme, cumin, rosemary, garlic, clove, ginger, cinnamon, and *Zataria multiflora*, against spoilage and pathogenic bacteria when used in edible films or coatings [10,11,23,24,25,26]. The present study covers the antimicrobial and sensory effects of wheat-gluten-based ECs containing EO obtained from PVR, which has not been studied before in an EC composition.

### 1.2. Pistacia vera *L.* Resin and Essential Oil

The genus *Pistacia* stands out among the Anacardiaceae family for its large number of species and varieties of plants. These species are prevalent throughout the Mediterranean and Middle East regions. Many studies have investigated the traditional medicinal features of *Pistacia vera* L. tree resin (PVR) and its EOs [27,28,29,30]. However, there is no research that includes the preparation of ECs using PVR and its EO in assessing antimicrobial activities against *Listeria monocytogenes* and *Salmonella* Typhimurium in CBFs. PVR is obtained from pistachio nut trees and is mainly a tree exudate secreted from the branches and bodies of damaged tree parts. It can be defined as gum or resinous exudate. PVR and its EO have several positive health effects and antimicrobial properties, making them potentially valuable additives for developing new antimicrobial edible films and coatings [30]. 

### 1.3. Scope and Purpose of the Study

The central hypothesis of this research is that ECs containing natural antimicrobial substances can prevent or slow down microbial growth within food material and keep consumers safe by inhibiting pathogens, without altering the sensory characteristics of CBFs adversely. In this study, wheat-gluten–PVR-based edible coatings (EC) were prepared with various concentrations of EO (0.5, 1, 1.5, and 2% *v/v*) to observe their antimicrobial and sensory effects on CBFs. Total bacteria count (TBC), *L. monocytogenes*, and *S.* Typhimurium counts were taken over the course of 12 days of storage at 8 °C. For the sensory analysis, a five-point hedonic scale was used to observe the effect of ECEO (2%) on the organoleptic properties of raw and grilled CBFs on the fifth day of storage (except for “taste” it was tested on the first day of storage).

## 2. Materials and Methods

### 2.1. Obtaining Pistacia vera *L.* Tree Resin and Essential Oil Extraction

**Figure 1:**
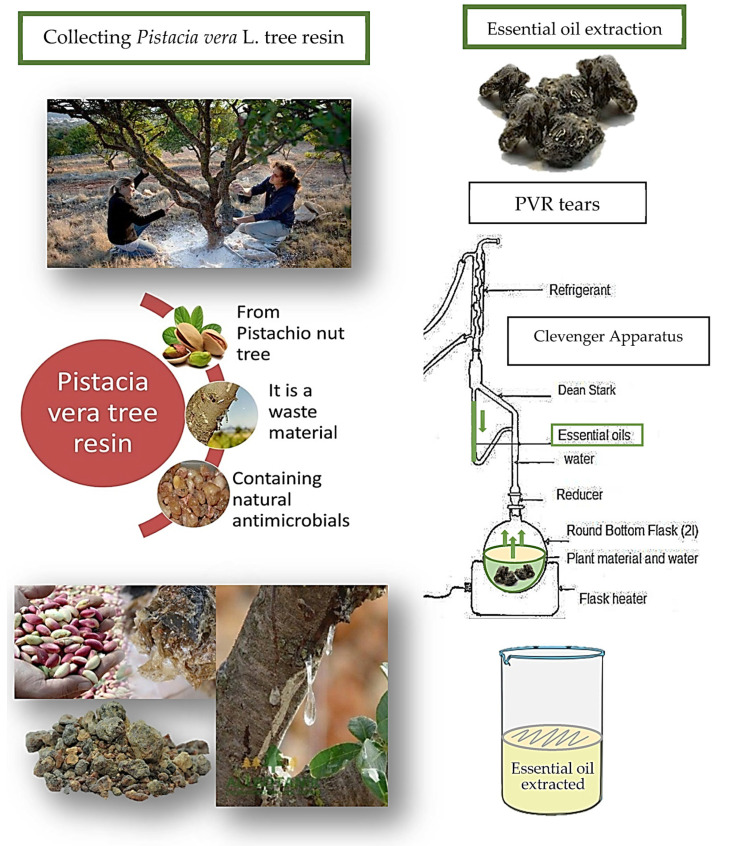
*Pistacia vera* L. resin (PVR) collected from pistachio nut trees and essential oil extraction from PVR using the Clevenger apparatus.

PVR was obtained directly from *Pistacia vera* L. trees in the villages around Gaziantep province in Turkey. It was collected during the fall and spring months of the year and stored in dark and cool cabinets (20 ± 2 °C) for further use. The EO of PVR was obtained using the Clevenger hydrodistillation (steam distillation) method [31] using the Clevenger apparatus (Inter Lab, Adana, Turkey). Ground PVR (150.0 g ± 1.5 g) was placed in an empty flask (2 L), and 1.5 L of double distilled water was boiled to obtain steam for distillation. In the next step, the sample was hydrodistilled for five hours (Figure 1) and kept in small (20 mL) sterile dark bottles at 4 °C until further use.

### 2.2. Preparation of Bacterial Strains and Contamination of Chicken Breast Fillets

*Salmonella enterica* subsp. *enterica* ser. Typhimurium ATCC 14028 and *Listeria monocytogenes* ATCC 35152 were obtained from the American Type Culture Collection (Rockville, MD, USA). The stock cultures of the bacteria were maintained on brain–heart infusion agar (BHIA, Merck, Darmstadt, Germany) slants at 4 °C. The bacterial cultures for the experiments were subcultured twice by inoculating them in 5 mL of tryptic soy broth (TSB, Merck, Darmstadt, Germany). The inoculated broths were incubated at 35 °C for 24 h. After incubation, 200 μL of bacterial culture was inoculated in fresh TSB and incubated at 35 °C for 24 h. Then, the cultures in the growth phase of an approximately 1 × 10^4^ colony-forming unit (cfu)/mL were used to inoculate the chicken samples. 

The CBFs were bought from local poultry markets, weighing nearly 145 g (±0.5 g) each. For further tests, the control groups of the food samples without contamination and coating were separated and stored at 8 °C. A dip-inoculation method was used to contaminate the CBFs separately with each pathogen. For this purpose, a stock inoculum solution with a targeted inoculation level of about 10^4^ cfu/mL was prepared by transferring 24 h TSB cultures of *S.* Typhimurium or *L. monocytogenes* into a 500 mL Ringer’s solution (1% *v/v*). Then, the CBFs were chopped into chicken cubes (30 g, 2 × 2.5 × 2 cm) and immersed in the stock solutions. The mixture was shaken for 1 min by hand to distribute the inoculum homogenously to the chicken breast samples. They were then kept in a biological safety cabinet (NuAire model Nu-425-200, Plymouth, MN, USA) at 22 ± 2 °C for 1 h. The inoculated chicken breast samples were then placed on rough filter paper under aseptic conditions to drain the excess solution [32].

### 2.3. Preparation of Edible Coatings and Application on Chicken Breast Fillets

**Figure 2:**
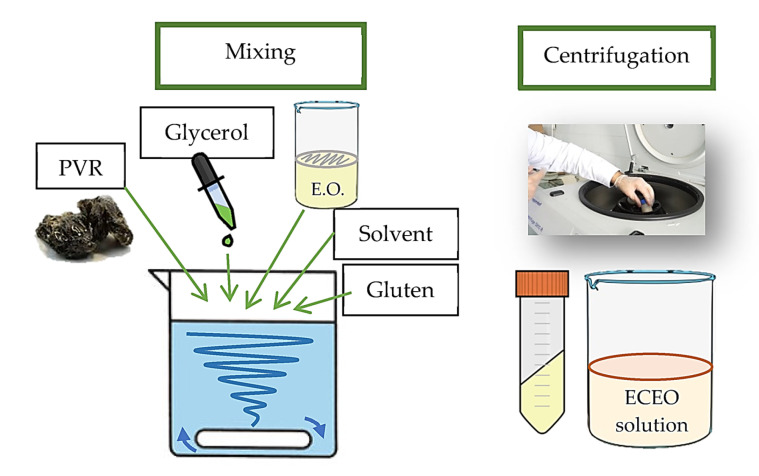
Preparation of ECEO solution by mixing and centrifugation.

The edible-coating (ECEO) solution based on wheat gluten–PVR with its EO was prepared using the film-forming dispersion method. First, the collected PVR was ground using a mortar. To generate 100 mL of the coating solution, the PVR (1.5% *w/v*) and vital wheat gluten (Tereos Vital Gluten, Belgium EU) (4.5% *w/v*) were dissolved in absolute ethanol (45% *v/v*) and mixed using a magnetic stirrer (Heidolph, MR-Hei standard, Germany). As a plasticizer, glycerol (ACS grade CAS 56-81-5, Millipore Merck, Darmstadt, Germany) (1.5% *v/v*) was added to the solution containing PVR and gluten. The pH was adjusted to 11.0 by adding ammonium hydroxide (1 M) dropwise to the solution while mixing with a magnetic stirrer. Then, the mixture was heated to 75 °C and mixed for 30 min in the magnetic stirrer. EO was added to the EC solution at several concentrations (0.5, 1, 1.5, and 2% *v/v*), and the coating solution was completed to 100 mL by adding distilled water. The mixture was centrifuged at 4950× *g* for 6 min at 18 °C (Eppendorf 5810R, Hamburg, Germany) (Figure 2). 

**Figure 3:**
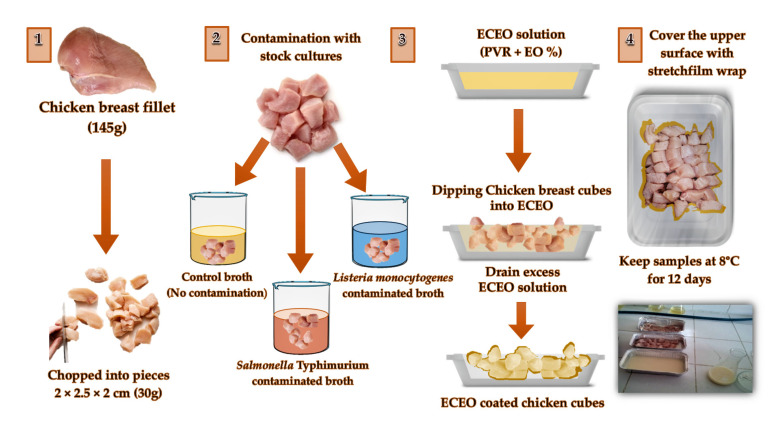
Contamination and application of ECEO coating solution on chicken breast fillet cubes. Numbers 1–4 designates the process steps. 1: Preparation of chicken breast fillet cubes, 2: Contamination with stock cultures, 3: Application of ECEO solution, 4: Storage of samples.

After centrifugation, the supernatant part (250.0 ± 0.5 mL of clear film dispersion) was poured into polytetrafluoroethylene (Teflon) plates and stored for 24 h at 8 °C for degassing. The CBF cubes were immersed in the coating solution with the dipping method (20 mL solution/kg of chicken breast fillets) and kept there for 1 min (Figure 3).

In the next step, the coated chicken breast cubes were placed on greaseproof paper in a vacuum oven at 25 °C for 5 min. The excess coating solution was drained, and the excess ethanol was evaporated. The ECEO solution composition and treatments for the samples are shown in Table 1. The ECEO-coated samples and controls (30.0 ± 0.5 g each) were packed into foam trays wrapped with low-density polyethylene stretch film and stored at 8 °C in a refrigerator without a modified atmosphere for 12 days. During storage, the samples were taken for microbial analysis on the 1st, 3rd, 5th, 7th, 10th, and 12th days of storage. 

### 2.4. Microbiological Analysis

The TBC and total numbers of *L. monocytogenes* and *S.* Typhimurium in the CBF were analyzed on the 1st, 3rd, 5th, 7th, 10th, and 12th days of storage. For each sampling day, the 25 g chicken samples were homogenized in 225 mL 0.1% buffered peptone water using a stomacher (Seward, 400 Circulator, Worthing, UK) for 1 min. Homogenized samples were then serially diluted using 0.1% buffered peptone water. They were spread-plated on plate count agar (for TBC, Merck Millipore 105463, Burlington, MA, USA), Wilson-Blair bismuth sulfite agar (for *S.* Typhimurium count, Merck Millipore 100191, Burlington, MA, USA), and Oxford base agar with the Listeria Selective Supplement (for *L. monocytogenes* count, Merck Millipore 107004 with 107006, Burlington, MA, USA). All plates were incubated at 37 °C for 48 h [33,34,35]. The numbers of presumptive colonies on each sampling day were recorded as log colony-forming units/g sample (log cfu/g).

### 2.5. Sensory Analysis

For the sensory evaluation of the CBF chopped as cubes and coated with ECEO (2%), the primary purpose was to investigate whether the ECEO coating was effective on the organoleptic properties negatively. Therefore, for the sensory analysis, uncontaminated samples were used, and the entire experimental design was divided into two sections: raw and grilled CBF cubes with and without coating. The testing samples and treatments applied are given in Table 2. 

A panel of graduate students and professors (*n* = 30) from the Food Engineering Department of Gaziantep University, Turkey, was invited to evaluate the sensory properties of the chicken breast cubes. All panelists were first passed through an induction process and also trained in the sensory properties that would be tested. They were asked to assess the smell, texture, and general acceptance of the raw (UCR and CR) and grilled (UCG and CG) chicken breast samples according to their appearance. A hedonic scale ranging from 1 to 5, with “1” standing for “disliked very much” and “5” standing for “liked very much”, was used for the assessment. The “taste” evaluation was performed only on grilled samples, while all other parameters were examined on raw and grilled samples, according to Watts et al., (1989), ASTM 1992, and Lawless et al. [36,37,38]. The results were recorded anonymously, and three replicates were given to the panelists to obtain the averages for each parameter scoring. Spider-web graphs were used to distinguish the parameters and scores.

**Figure 4:**
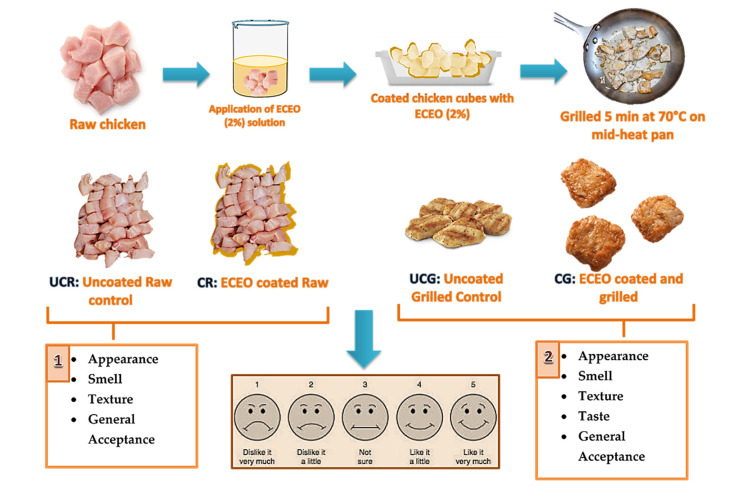
Preparation of raw and grilled CBF cubes coated with ECEO (2%) for sensory analysis. Numbers 1 and 2 designates the test parameters applied on smples for sensory analysis. 1: Test parameters scored by panelists for raw CBF cubes, 2: Test parameters scored by panelists for grilled CBF cubes.

Half of the raw chicken breast cubes were coated with the ECEO (2%) coating for sensory analysis, and the other half was kept uncoated. All of the samples were kept at 8 °C for five days. Before five days, no significant changes in sensory properties were detected in the preliminary studies. To evaluate the “taste” property, on the first day of storage, half of the samples were grilled on a kitchen pan at around 70 °C, measured central temperatures of CBF cubes for 5 min, and served to the panelists for scoring (Figure 4). Sensory properties other than “taste” were evaluated on the fifth day of storage.

### 2.6. Statistical Analysis 

All analyses were performed with three replications for each sample. The results were reported as the means ± standard errors. All data were analyzed using SPSS 22.0 for Windows (IBM SPSS, Chicago, IL, USA) and compared using analysis of variance (ANOVA), and the main effects were considered significant at the *p* < 0.05 level. In addition, a paired samples *t*-test was applied for the sensory analysis scores between the coated and uncoated samples for the same parameters scored.

## 3. Results and Discussion

### 3.1. Antimicrobial Effects of PVR Edible Coating on Chicken Breast Fillets

**Figure 5:**
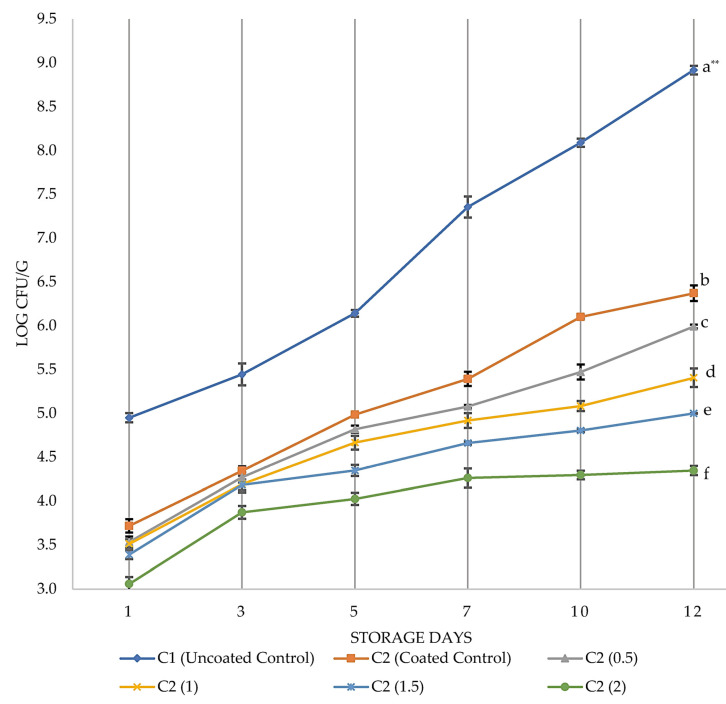
Growth of total bacteria count on controls C1 and C2 with different essential-oil concentrations during storage for 12 days at 8 °C. Data shown are means ± standard errors. Samples significantly different from each other at the end of 12 days of storage according to Duncan post hoc tests were C1, C2, C2_(0.5)_, C2_(1)_, C2_(1.5)_, and C2_(2)_. ** Different small letters on the same day indicate significant difference at *p* < 0.05 level.

Figure 5 shows the results of the TBC counts in the coatings C1, C2, and C2_EO_ (containing EO from 0.5 to 2.0%). The TBC of CBF without coating (C1) was about 4.9 log cfu/g on the first day and increased to 8.9 cfu/g after 12 days of storage, similar to previous studies [3,39,40,41,42,43,44,45,46]. The application of ECEO on the chicken with various EO concentrations (C2) significantly suppressed the growth of TBC during storage periods (*p* < 0.05). After 12 days of storage, the TBC was increased in C1 by 4 logs and in C2 by 2.7 logs cfu/g. Increasing the amount of EO in the ECEO solution increased the antimicrobial effect on TBC. At the end of 12 days of storage, all ECEO samples decreased the TBC numbers by a significant level compared to C1 samples (*p* < 0.05). Meat and meat products should contain no more than 7 log CFU/g of TBC; according to the Food and Agricultural Organization (FAO) [47], the acceptable limit for TBC in poultry is below <6 log cfu/g [3,48,49]. In our study, the coated samples have successfully kept the TBC numbers at the allowed limits while the uncoated control sample C1 reached this limit on the seventh day.

In the present study, the ECEO coatings showed a bacteriostatic effect on TBC. The reasons for the cause of this can be explained by both the physical barrier properties of the coating material (wheat gluten and PVR) and the antimicrobial effect of EO present in the EC composition. Generally, ECs made from wheat gluten greatly limit water vapor transfer through the coating to the food surface, causing lowered water activity on the surfaces of the food materials coated [50]. The hydrophobic-coating compositions, such as wheat-gluten-based coatings, showed antimicrobial effects against common spoilage microorganisms found in poultry products. Similar to gluten, chitosan, and carboxymethyl cellulose also have hydrophobic interactions. Studies show that they succeeded against lipid oxidation and other enzymatic spoilage but failed against microbial spoilage when used alone in the EC composition. Various EOs have been used to increase the antimicrobial effectiveness of these coatings and prolong the shelf life of poultry by inhibiting the growth of common spoilage microorganisms, which include psychrotrophic bacteria, lactic acid bacteria, *Pseudomonas, Campylobacter,* and *Enterobacteriaceae* [23,44,48]. Most studies related to the antimicrobial effects of EOs emphasize that EOs may change the cell wall permeability and intracellular alterations, leading to cell death [4,11,13,44,49,51,52,53]. Alma et al. [30] observed the chemical composition and antimicrobial activity of the EO of PVR against 13 bacteria and three yeast species. In the minimum inhibitory concentration (MIC) tests, EO obtained from PVR inhibited nine of the 13 bacteria and all yeasts. In a related study, Ghalem and Mohamed [27] investigated the antimicrobial activity of the EO of PVR against *E. coli*, *Proteus*, and *S. aureus*, and EO was found to inhibit the growth of all bacteria.

In addition to the EO of PVR and wheat gluten, other ECs containing different EOs have antimicrobial effects on CBFs. Garavito et al. used guar gum, nisin, and oregano oil as EC ingredients for application on CBFs. Similar to our present study with EO of PVR, nisin- and oregano oil-containing samples showed a bacteriostatic effect on TBC during 16 days of storage at 4 °C [4]. Bazargani-Gilani et al. [40] investigated the antimicrobial effect of pomegranate juice and chitosan coating (which is hydrophobic, as is the wheat gluten used in our study) enriched with *Zataria multiflora* essential oil (ZEO) on chicken meat stored at 4 °C. Samples containing ZEO and chitosan significantly lowered the number of TVC on each sampling day during 20 days of storage. In a related vein, Fernández-Pan et al. [3] researched the antimicrobial efficacy of whey protein isolate (WPI) coating with oregano and clove EO on CBFs stored at 4 °C. The WPI coating containing 20 g/kg oregano EO was the most effective, having 2 log reductions against aerobic mesophilic bacteria and 1 log reduction against psychrotrophic bacteria and *Enterobacteriaceae*. Generally, the results of TBC are in concordance with studies that included EO-blended ECs conducted with CBFs [24,44,46,48].

**Figure 6:**
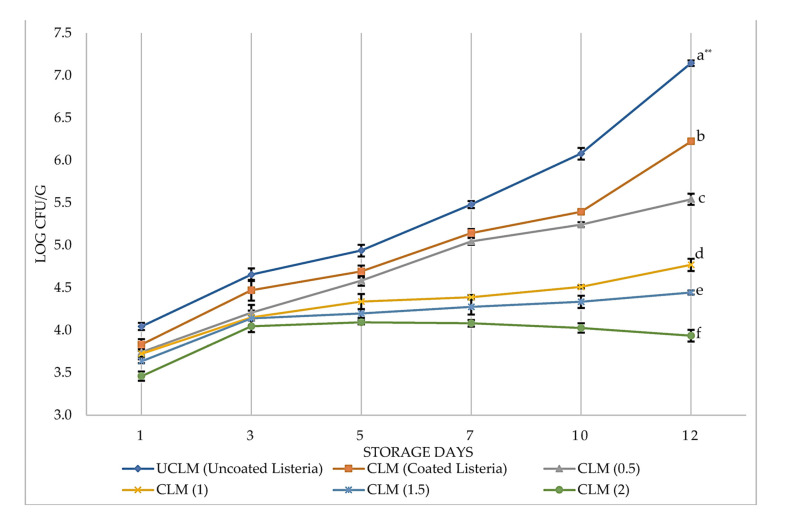
Growth of *L. monocytogenes* on the UCLM and CLM with different EO concentrations during storage for 12 days at 8 °C. Data shown are means ± standard errors. Samples significantly different from each other at the end of 12 days of storage according to Duncan post hoc tests were UCLM, CLM, CLM_(0.5)_, CLM_(1)_, CLM_(1.5)_, and C2_(2)_. ** Different small letters on the same day indicate significant difference at *p* < 0.05 level.

**Figure 7:**
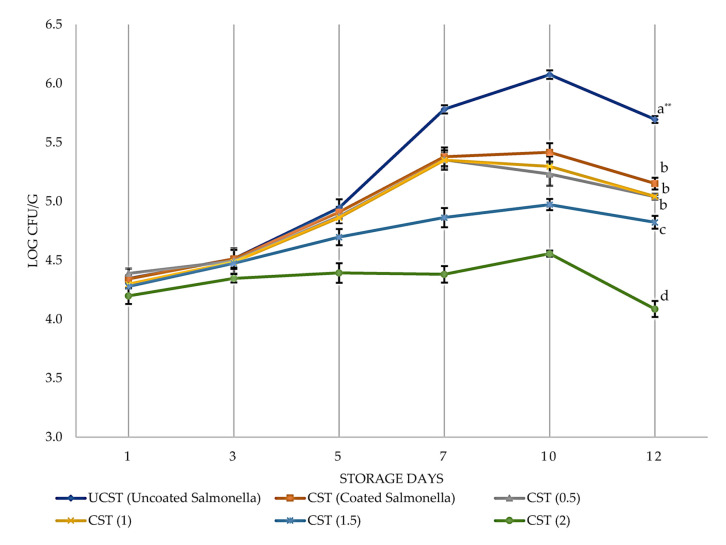
Growth of *S.* Typhimurium on the UCST and CST with different essential oil concentrations during storage of 12 days at 8 °C. Data shown are means ± standard errors. Samples significantly different from each other at the end of 12 days of storage according to Duncan post hoc tests were; UCST; (CST, CST_(0.5)_, CST_(1)_); CST_(1.5)_; and CST_(2)_. ** Different small letters on the same day indicate significant difference at *p* < 0.05 level.

In the present study, *L. monocytogenes* counts were obtained in coated and uncoated CBF contaminated by *L. monocytogenes* (Figure 6). The EO-added coating (CLM including various EO concentrations) had a higher antimicrobial effect against *L. monocytogenes* than the uncoated *L. monocytogenes* (UCLM) (*p* < 0.05). The antimicrobial activity increased with increasing EO concentration, while *L. monocytogenes* in coated *L. monocytogenes* (CLM) reached 6.2 log cfu/g. Furthermore, *L. monocytogenes* increased to only 3.9 log cfu/g in CLM_(2)_ at the end of 12 days of storage (*p* < 0.05). The number of *L. monocytogenes* in UCLM samples increased by 3.1 logs; however, the number in CLM samples increased by 2.4 logs during storage. The antimicrobial activity of the coating increased significantly with EO addition, with even CLM_(0.5)_ coating lowered the *L. monocytogenes* count (*p* < 0.05). The highest antimicrobial effect was seen in CLM_(2)_, which inhibited the growth of *L. monocytogenes*. The antimicrobial activities of CLM_(1)_, CLM_(1.5)_, and CLM_(2)_ against *L. monocytogenes* were not significantly different (*p* > 0.05) from each other throughout the duration of the storage period. A previous study showed that the MIC of the EO of PVR against *L. monocytogenes* was 0.25% [54]. Therefore, all the coated samples with EO concentrations above the MIC of 0.25% should be effective against *L. monocytogenes* growth. By looking at similar growth trends of TBC (Figure 6) and *L. monocytogenes* (Figure 7) counts, one can say that *L. monocytogenes* adapted well to highly competitive flora during storage. However, *L. monocytogenes* growth in the CLM samples including EO in the coating composition was significantly limited. Similar results in the subsequent studies showed that *L. monocytogenes* was susceptible to EO-added coatings [11,44,48,52,55,56]. 

In one particular study, Abbasi et al. [11] observed the antibacterial effects of fortified nanoemulsions of starch-based ECs, including ZEO, on chicken meat. The results showed that uncoated control samples reached 11.42 log cfu/g from 4 logs for the initial *L. monocytogenes* number, where the most effective coating nanoemulsion of ZEO with cinnamaldehyde reached only 6 log cfu/g at the end of 20 days. In another study, Shekarforoush et al. [44] found that *L. monocytogenes* numbers did not change significantly (*p* > 0.05) in ready-to-barbecue chicken meat coated with chitosan and oregano EO and stored at 8 °C with 4.7 log cfu/g. Unlike our storage period, their results covered only the first 3 days of storage. During the same duration of storage, ECEO samples had a slight increase of 0.5 logs from an initial 3.5 log cfu/g to 4.0 log cfu/g on the 3rd day.

Generally, adding EO or another antimicrobial agent increases the effectiveness of the preservation potential of ECs against pathogenic bacteria. Raeisi et al. [52] studied the combined effects of rosemary, cinnamon EOs, and nisin and found a greater inhibitory effect on *L. monocytogenes* during the storage of chicken meat. Nouri Ala et al. [48] reported that bioactive carboxymethyl cellulose coatings containing *Ziziphora clinopodioides* (ZEO; 0.25 and 0.5%) and *Mentha spicata* (MEO; 0.5%) EOs applied to CBFs caused *L. monocytogenes* numbers to increase from 5 log cfu/g to 7.55 and 7.83 logs for negative and positive controls, respectively. In the research of Souza et al. [55], bio-nanocomposite edible films containing ginger EO had antimicrobial effects against *L. monocytogenes* and other pathogens in chicken breast samples. Janes et al. [56] studied the effect of zein propylene glycol film containing nisin and calcium propionate as antimicrobial agents on chicken meat against *L. monocytogenes* during 8 °C and 4 °C storage, finding that *L. monocytogenes* growth was suppressed by 5.4 logs on day 8 compared to uncoated control samples stored at 8 °C. Our present study showed a 4-log decrease in CLM_(2)_ compared to UCLM on day 12. One can say that ECs incorporated with antimicrobial agents rather than EOs (nisin, calcium propionate, etc.) showed a higher decrease in *L. monocytogenes* numbers. However, ECs containing other EOs, even combined with other antimicrobial agents, have similar antimicrobial effect of ECEO-coated samples.

In the present study, the uncoated *S.* Typhimurium (UCST) samples from an initial microbial load of 4.3 log cfu/g reached 6.1 log cfu/g at 10 days of storage (Figure 7). The coating containing EO 1.5% and higher showed significant inhibition against the growth of *S.* Typhimurium (*p* < 0.05), suppressing their growth by approximately one log at the end of storage duration. A previous study also showed that the MIC of EO obtained from PVR against *S.* Typhimurium was 1.5% (*v/v*) [54]. At the end of 12 days of storage, *S.* Typhimurium numbers in the samples CST, CST_(0.5)_, and CST_(1)_ were not significantly different (*p* > 0.05), while CST_(2)_ had a significant decrease in the *S.* Typhimurium numbers. These samples had results around 5 log cfu/g at day 12, where CST_(1.5)_ and CST_(2)_ were more effective than the other coated samples having 4.8 log cfu/g and 4.1 log cfu/g, respectively. As expected, the most effective coating was CST_(2)_, with significantly lower *S.* Typhimurium numbers during storage (*p* < 0.05). 

In our study, the *S.* Typhimurium count fluctuated during storage at 8 °C. Bacterial growth increased dramatically in the first 5 days, slowed down in the following days, and decreased after the 10th day of storage. The main reason for the decrease in *S.* Typhimurium count in all samples after the 10th day of storage may be because *S.* Typhimurium, as a mesophilic bacteria, could not compete with the mainly psychrophilic bacterial flora in the samples at refrigeration temperatures [57]. This uncompetitive characteristic has been seen in storage temperatures of 4 °C dramatically in the following studies. Nouri Ala et al. [48] have reported that the TBC of uncoated samples increased to 9 log cfu/g on the 10th day of storage, and the most effective coating with EOs increased to 4.2 log cfu/g from the initial number of 3.55 log cfu/g. Furthermore, carboxymethyl cellulose coatings with several EOs decreased *S.* Typhimurium by 3 logs at the end of 13 days of storage at 4 °C. This direct decrease, differing from our present study in terms of *S.* Typhimurium numbers, can be explained by lower storage temperatures than our storage conditions. *S.* Typhimurium is a well-known bacterium with uncompetitive growth characteristics at lower refrigeration temperatures, such as 4 °C [48].

In another vein, de Moraes Pinto et al. [5] observed the microbial and quality properties of CBFs treated with sodium alginate ECs containing oregano and curcumin EOs. According to the *S.* Typhimurium count results, all samples containing EOs had approximately 2 log decreases during a 7-day storage period at refrigerated temperatures. Unlike our results, they observed a continuous decrease in *S.* Typhimurium counts in all samples during the entire storage period. All samples with coatings had significantly lower numbers than the control samples without any significant difference from each other. 

Goswami et al. [58] used a pea-starch coating with thyme EO to research the antimicrobial effects against pathogens and spoilage bacteria found in chicken breast meat. They found that total aerobic counts increased from 4.7 to 7.1 log cfu/g during a 12-day storage period at 4 °C in *Salmonella*-inoculated control samples. EO-added samples had similar results, ranging from 4.0 to 7.2 log cfu/g. At the same time, *S.* Typhimurium count results showed a decrease in control samples from 5.2 to 4.2 log cfu/g, whereas EO-added samples had a significantly higher decrease from 4.3 to 2.2 log cfu/g. Their results were similar to our TBC numbers but different from the *S.* Typhimurium count results. Since their storage was at 4 °C, this difference is expected due to the *S.* Typhimurium growth characteristic at lower temperatures mentioned above. As a general view, in the case of *S.* Typhimurium growth, storage temperature changes the growth trend with a high impact since in our experimental conditions simulating refrigerator temperatures at 8 °C limited the growth during the storage period. Studies having 4 °C storage temperature discussed above have similar results in decreasing *S.* Typhimurium numbers from day 1. Further investigation of different storage temperatures with wheat-gluten–PVR-based ECs with various concentrations of EOs should be considered in future studies. 

### 3.2. Sensory Analysis of Chicken Breast Fillets with PVR Edible Coating

**Figure 8:**
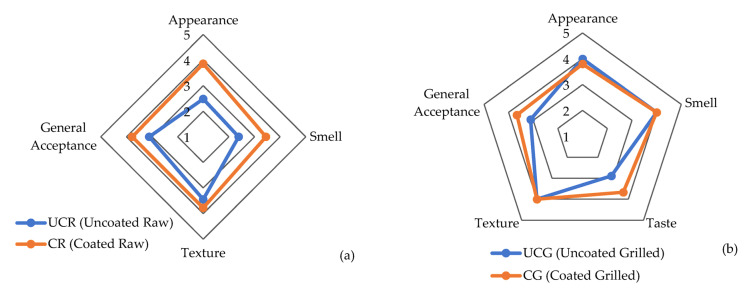
Sensory analysis scores: (**a**) uncoated and coated raw chicken breast cubes and (**b**) uncoated and coated grilled chicken breast cubes with taste parameter at the end of 5 days of storage at 8 °C. Data shown are means of scores given. Numbers 1–5 designates score axis of hedonic scale while points indicate the means of scores given by panelists to each sensory parameter.

The results of the sensory analysis for raw CBF cubes according to quality parameters of appearance, smell, texture, and general acceptance are shown in Figure 8a. The appearance, smell, and general acceptance of CR were scored significantly better than for the UCR samples (*p* < 0.05). In other words, C2_(2)_ coating maintained the sensory properties of raw CBF cubes at a significantly higher acceptance level (*p* < 0.05) than UCR, except for texture.

Furthermore, the results of the grilled CBF cubes tested for quality parameters of appearance, smell, texture, taste, and general acceptance are given in Figure 8b. Again, both of the samples, UCG and CG, had similar and higher scores, meaning C2_(2)_ coating did not alter the appearance, smell, and texture of grilled chicken breasts (*p* > 0.05). However, the taste and general acceptance of the CG samples were significantly higher than the UCG samples (*p* < 0.05). Among all the parameters, the smell is crucial for a suitable EC application on food material. Coated raw samples had a significantly lower smell score than uncoated raw CBF samples but this difference was not significant in the grilled samples (*p* > 0.05). In addition, the “taste” of the CG samples was scored significantly higher than that of the UCG samples (*p* < 0.05), meaning that C2_(2)_ coating containing EO of PVR can be used for this food material, as it keeps organoleptic properties at reasonable levels.

Studies include sensory analysis of ECs when EOs are considered as suspects for causing changes in the organoleptic properties of food materials [59]. Since the present study is the first to conduct a sensory evaluation of EOs obtained from *Pistacia vera* L. resin, comparisons of the sensory evaluations were conducted with antimicrobial ECs containing other EOs. Accordingly, it is worth noting that Panahi et al. [60] obtained odor results similar to our study, with lower scores of uncoated controls than samples coated using sodium alginate incorporated with *Ferulago angulate*, Boiss EO, and nisin during 12 days of storage. Similarly, Bazargani-Gilani et al. [40] investigated a pomegranate juice-added chitosan coating enriched with ZEO on chicken breast meats over 20 days of storage. Pomegranate juice-treated samples showed significantly higher scores than all the control groups. Before 5 days of storage, the odor of samples treated with ZEO was higher than the control, but after 5 days, off odor due to microbial spoilage occurred. The results obtained before 5 days of storage showed that ZEO-treated coatings improved the odor of chicken breast samples, which suits our findings for the smell.

In a study with very similar odor property results, Garavito et al. [4] developed an EC of guar gum and isolated soy protein enriched with oregano EO. According to the sensory evaluation during 10 days of storage, all sensory parameters of the coated samples were kept at acceptable levels during the first 6 days of storage. The odor of uncoated samples decreased significantly in comparison to the coated samples on day 6, which is similar to our results on the fifth day (*p* < 0.05).

Nouri Ala et al. [48] formed carboxymethyl cellulose coatings that are hydrophobic, similar to the wheat gluten used in this study, with several plant-based EOs (ZEO and MEO), and recorded the lowest sensory scores for the uncoated chicken fillets. In addition, the coating did not adversely affect the sensory characteristics of the chicken meat samples. In another study, the taste of the chicken samples was unaffected by using chitosan film combined with oregano EO, which also increased the shelf life of chicken fillets by 14 days while maintaining acceptable sensory attributes [41]. 

Yousefi et al. [61] had decreased sensory properties during storage of 16 days at 4 °C for the lactoperoxidase system-alginate-coated chicken breasts. On day 0, all samples had high sensory scores of 8/10, but on day 16, only the coated samples had acceptable scores. According to the panelists, the results of the products were unacceptable for uncoated samples at the end of the storage period. 

For ECs applied to food materials, sensory properties are crucial in influencing consumer choices and decisions. While preserving food materials from harmful effects, the coating should keep the sensory properties “acceptable” to consumers. The present and previous studies showed that when ECs and their bioactive components are used at appropriate levels, they have no adverse effects on the sensory properties of food materials. 

## 4. Conclusions

This research showed that *Pistacia vera* L. tree resin and its EO could be used to produce wheat-gluten–PVR-resin-based antimicrobial ECEO coatings on poultry to keep them safe from pathogenic bacteria and to protect sensory properties without any adverse alterations during refrigeration storage. The ECEO coating used in this research showed remarkable antibacterial properties at a 2% level of addition against *S.* Typhimurium and *L. monocytogenes,* which can be found in chicken meat products. In addition, the coating and its components had no adverse effects on the sensory properties of grilled CBFs. As the first study of *Pistacia vera* L. resin EO in an antimicrobial edible coating composition and sensory evaluation of CBFs, the ECEO coating may have high research and commercial potential. Therefore, it can be considered a feasible and reliable alternative for preserving CBFs without losing sensory parameters. On the other side, limited research in the literature makes comparing PVR and its EO with other coating materials and antimicrobial agents. Thus, further studies, including the physical and mechanical properties of PVR edible films and coatings, chemical compositional properties and behaviors in different mixtures, and applications to other food materials at various conditions, are worth discovering. 

## 5. Patents

Edible coating and its components used in this research are a part of the patented product licensed by the Turkish Patent Office (TPE), and the corresponding authors of this article reserve all intellectual property and commercial production rights. 

“Edible antimicrobial film produced from pistachio resin. This invention relates to a film with edible antimicrobial properties produced using pistachio resin (PVR) and the production method of this edible film. 24/04/2018, Patent Registration, National, Application No: TR2015/00217” by: National Patent given by Turkish patent office. 

## Figures and Tables

**Table 1:** ECEO application on chicken breast fillets, contamination of pathogens, and analysis.

Type of CBF	Edible Coating Application	Contamination of Pathogens	Microbial Count
C1	Uncoated CBF	No contamination	TBC
C2	Coating CBF with EC		
C2_(0.5)_ *, C2_(1)_, C2_(1.5)_, C2_(2)_	Coating CBF with ECEO		
UCLM	Uncoated CBF	*L. monocytogenes* contamination	*L. monocytogenes* count
CLM	Coating CBF with EC		
CLM_(0.5)_, CLM_(1)_ CLM_(1.5)_, CLM_(2)_	Coating CBF with ECEO		
UCST	Uncoated CST	*S.* Typhimurium contamination	*S.* Typhimurium count
CST	Coating CBF with EC		
CST_(0.5)_, CST_(1)_, CST_(1.5)_, CST_(2)_	Coating CBF with ECEO		

* Numbers on the subscripts indicate essential oil (EO) concentration in the EC, EC: edible coating, ECEO: edible coating containing EO.

**Table 2:** Sensory analysis of raw and grilled chicken breast fillet cubes with and without ECEO (2%) coating.

Name of the Sample	Treatment	Sensory Analysis
UCR	Uncoated raw CBF	Appearance, smell, texture, general acceptance, and taste (only for grilled samples)
CR	Coating raw CBF with ECEO (containing 2% EO)	
UCG	Uncoated CBF grilled for 5 min	
CG	Coating CBF with ECEO (2%) + grilled for 5 min	

## Data Availability

The data presented in this study are available on request from the corresponding author.
